# Consensus Sequence of 27 African Horse Sickness Virus Genomes from Viruses Collected over a 76-Year Period (1933 to 2009)

**DOI:** 10.1128/genomeA.00921-15

**Published:** 2015-09-10

**Authors:** A. Christiaan Potgieter, Isabella M. Wright, Alberdina A. van Dijk

**Affiliations:** a OIE Reference Laboratories for African Horse Sickness Virus and Bluetongue Virus, Virology Division, ARC, Onderstepoort Veterinary Institute, Onderstepoort, South Africa; b Biochemistry Division, North-West University, Potchefstroom, South Africa

## Abstract

We announce the complete consensus genome sequence of 27 African horse sickness viruses, representing all nine African horse sickness virus (AHSV) serotypes from historical and recent isolates collected over a 76-year period (1933 to 2009). The data set includes the sequence of the virulent Office International des Epizooties AHSV reference strains which are not adapted to cell culture.

## GENOME ANNOUNCEMENT

African horse sickness (AHS) was first identified as a disease in horses in South Africa in 1891 and the viral nature of its etiological agent, African horse sickness virus (AHSV), was established in 1900 (1). The virus is transmitted by hematophagous *Culicoides* midges (2, 3) and can cause mortalities in up to 95% of fully susceptible horses. AHS is endemic in most of sub-Saharan Africa, occurs sporadically in North Africa, Mediterranean countries, and the Middle East, and a few outbreaks have been recorded in India and Pakistan. AHSV is a double-stranded RNA (dsRNA) virus with ten genome segments that belongs to the genus *Orbivirus*, family *Reoviridae*. Nine serotypes of the virus have been distinguished (4, 5).

Currently, the AHSV reference strains of the Office International des Epizooties (OIE) used at the ARC-Onderstepoort Veterinary Institute (OVI), South Africa, consist of at least one pathogenic isolate of each serotype from AHS outbreaks between 1955 and 1963 (6). These virulent reference viruses are used as challenge viruses during vaccine trials. Tissue culture–adapted progeny of these viruses and guinea pig sera raised against them are widely used as diagnostic reagents for serotyping AHSV isolates and to determine the immunity status of horses before and after vaccination (6).

The histories of the viruses used in this study (Table 1) were compiled from ARC-OVI specimen collection records from 1958 to 2009, records of vaccine trials in horses, personal communication with B. J. Erasmus, and the literature (4, 6–8). For this project, the virulent OIE reference strains and neurotropic vaccines were propagated by intracerebral injection of 2-day-old Swiss white mice, and the recent field isolates of AHSV were propagated in either BHK-21 or Vero cells. Extraction and purification of dsRNA, sequence-independent genome amplification, Roche 454 sequencing, and *de novo* sequence assembly were done as described previously (9).

**TABLE 1:** History of 27 AHSV isolates from a 76-year period (1933–2009)

AHSV serotype	Original isolate	Isolate no. or yr of isolation	Passage level^a^	Origin	Accession no.
History of the current OIE reference strains of African horse sickness virus used at ARC-OVI					
1	HS 61/61^b^	HS 29/62^c^	2S	Nelspruit, South Africa	KF859986 –KF859995
2	Unknown	HS 82/61^c^	3S	South Africa	KF859996–KF860005
3		HS 13/63^c^	4S	Malmesbury, South Africa	KM886354–KM886363
4	HS 47/58 (Specimen 341)	HS 32/62^c^	1S	Zimbabwe	KM609465–KM609474
5	FR (Fourie)	HS 30/62^c^	2S	South Africa	KM886344–KM886353
6	HS 09/58 (Mule 3858)	HS 39/63	1S	Kaalplaas, South Africa	KF860006–KF860015
6		HS 02/75^c^	4S	South Africa	KP009741–KP009750
7	HS 59/61	HS 31/62^c^	2S	Kaalplaas, South Africa	KF860016–KF860025
8		HS 10/62^c^	2S	Kenya	KF860026–KF860035
9		HS 90/61	4S	Chad (Fort Lamy)	KF860036–KF860045
History of recent field isolates of African horse sickness virus					
1	HS 21/07		Unknown	South Africa	KP009621–KP009630
2	HS 90/07		2Vero	South Africa	KP009631–KP009640
3	HS 73/08		Unknown	South Africa	KP009641–KP009650
4	HS 128/06		1Vero	South Africa	KP009651–KP009660
5	HS 28/08		Unknown	South Africa	KP009661−KP009670
6	HS 04/08		Unknown	South Africa	KP009671−KP009680
7	HS 23/08		Unknown	South Africa	KP009681−KP009690
8	HS 29/00		1S,5BHK	South Africa	KP009691−KP009700
8	HS 83/04		3Vero,1BHK	South Africa	KP009721−KP009730
9	HS 27/08		Unknown	South Africa	KP009701−KP009710
9	HS 72/08		Unknown	South Africa	KP009731−KP009740
9	HS145/09		Unknown	South Africa	KP033466−KP033475
History of the historic neurotropic strains of African horse sickness virus					
1	1180	1933	100+ in adult mice	South Africa	KP009711−KP009720
3	L	1940	100+ in adult mice	Ladysmith, South Africa	KP009761−KP009770
4	Vryheid	1938	100+ in adult mice	Vryheid, South Africa	KP009771−KP009780
5	Westerman	1936	100+ in adult mice	South Africa	KP009781−KP009790
7	Karen	1952	100+ in adult mice	Kabete, Kenya	KP009751−KP009760

^a^ The passage level refers to the final passage number before dsRNA extraction and sequencing.

^b^ The number after the / indicates the year of the original isolate and the number before the / indicates the number of the isolate during the particular year.

^c^ Virulent strains used to develop attenuated vaccines by serial passage in BHK-21 and selection of genetically stable large plaques on Vero cells (6, 7).

Here we announce the first complete consensus genome sequence of each of the nine original pathogenic OIE reference strains of AHSV prior to the adaptation of the viruses to cell culture (10), some of the neurotropic AHSV vaccine strains (4, 11), and recent isolates of all nine AHSV serotypes made at the ARC-OVI from 1998 to 2009 (Table 1). In all, we sequenced 10 OIE reference strains (including HS2/75 used for attenuation of AHSV6), 5 neurotropic historic vaccine strains, and 12 recent field isolates. This sequence data set of 27 AHSV strains is the first representing all nine AHSV serotypes from both historical and recent isolates collected over a long period of 76 years (1933 to 2009). These genome sequence sets should be useful for comparison with sequences from live vaccine strains that were derived from them and published sequences from cloned genome segments that are mostly incorrect due to cloning biases, such as the genome segment 2 (VP2) sequence set of all nine AHSV serotypes (12). The consensus genome sequence data set of this announcement has already allowed the development of group- and serotype-specific real-time RT-PCRs (13) and, more importantly, has been used to rescue AHSVs of all nine serotypes by reverse genetics, which verifies their correctness (14).

### Nucleotide sequence accession numbers.

The nucleotide sequences have been deposited in GenBank under the accession numbers listed in Table 1.
